# The Capacities of Primary Health Care in Hungary: A Problem Statement

**DOI:** 10.3390/ejihpe10010025

**Published:** 2019-12-23

**Authors:** Csaba Bálint

**Affiliations:** Institute of Regional Economics and Rural Development, Szent István University, H-2100 Gödöllő, Hungary; balint.csaba@gtk.szie.hu

**Keywords:** health care, primary health care, general practitioner, family paediatrician, vacant districts

## Abstract

In the establishment, development, and provision of equal access to the health care system, the operation of adequate primary health care is essential and has undergone significant transformation in the most developed countries over the past decades. The central and eastern European countries, including Hungary, are struggling with the disadvantages of the traditional model of primary health care, based on independent general practitioner and family paediatrician practices: the ability of the system is extremely limited to meet emerging needs and is facing a chronic human resource crisis. In the current study, the functions, legislation, and challenges of the Hungarian primary health care system, as well as the basic interrelations of the development of vacant general practitioner and family paediatrician districts were examined, and the government measures for the sake of solving the occurrence of the vacancy and improving access in the lagging areas. (The situation of the other fields of primary health care—e.g., dental care, child care officer care, etc.—was not subject of the analysis.). The basic characteristics of the vacant districts (type by supplied age group, bounding region, population size, length of vacancy) were primarily examined by the analysis of categorical and metric variables, with the use of cross-tabulation and nonparametric correlation, while the discovery of soft interrelations was supported by an expert interview conducted with the professionals of the Primary Health Care Department of the National Health Care Services Centre. In Hungary, the fundamentals of primary health care are made up of the individual practices of general practitioners and paediatricians, and there is a growing concern about the permanent vacancy of the districts, and the fact that the system is less suitable for meeting the needs of the population. The ever-increasing number of vacant general practitioner and family paediatrician districts due to the growing shortage of professionals because of aging and emigration poses the burden of substitution on the physicians in existing practices, that concerns the access of more than a half million people to health care, almost 70 percent of which live in settlements with a population less than 5000 inhabitants.

## 1. Introduction

“Everyone has the right to a standard of living adequate for the health and well-being of himself and of his family, including food, clothing, housing and medical care and necessary social services”, as stated in the United Nations’ Universal Declaration of Human Rights. At the foundation of the World Health Organization in Geneva in 1946 the concept of health was defined: “Health is a state of complete physical, mental and social wellbeing and not merely the absence of disease or disability” [1]. The organization’s 1979 document “Global Strategy for health for all by the year 2000” separated the dimensions of health as follows [2]: biological health—the proper functioning of the body; psychic health—personal world view, basic behavioural principles, peace of mind; mental health—the ability of thinking clearly and consistently; emotional health—the ability for the recognition of emotions and their appropriate expression; social health—the health of building relationships with others.

Among the UN 2030 Sustainable Development Framework’s 17 Sustainable Development Goals (SDGs) the 3rd Goal (Health and Well-being—Ensuring Healthy Life and Creating Wealth for All People of All Ages) is specially health-centred. In line with the integrated concepts of sustainable development goals, the objectives of SDG3 are directly related to health and well-being, while influencing and being influenced by other development goals [3].

According to WHO’s 2016 report [4] “Framework on integrated, people-centred health services”, despite significant steps forward in human health and life expectancy in recent years, relative improvements have been uneven across and within countries. More than 400 million people worldwide have no access to basic health care. Where available, care is often too fragmented or of poor quality and consequently, in many areas, satisfaction with the health care system and health services remains low. In many countries, there are still significant problems with unequal geographical access to health services, lack of health workers, and weak supply chains. In order to achieve the 3rd Sustainable Development Goal (for all ages of healthy life and well-being), including Universal Health Coverage (3.8) objective, the UN requires countries to ensure that all people and communities have access to high quality, safe, and equitable health services.

In general, territorial differences are due to the reorganization of economic and social processes in space and time. The underdeveloped areas are characterized by unsatisfied internal needs, constantly declining services, devastating natural values, and conflicts from the former, and the lack of adaptability to global trends [5]. The level of social capital in a community also affects health indicators [6]. Although access to health care is an internationally recognized fundamental human right, access to health services is inadequate in many areas. There are many rural and remote communities around the world who, due to their unfavourable health status, would require high levels of health care, face significant (spatial and non-spatial) access barriers to health services [7].

In providing and improving access to care, primary health care plays an important role as the entry level of the care system for the following reasons [8]:

- The features of primary health care allow the health system to adapt and respond to a complex and rapidly changing world.
- With its emphasis on promotion and prevention, addressing determinants, and a people-centred approach, PHC has proven to be a highly effective and efficient way to address the main causes of, and risk factors for, poor health, as well as for handling the emerging challenges that may threaten health in the future.
- Universal health coverage (UHC) and the health-related sustainable development goals (SDGs) can only be sustainably achieved with a stronger emphasis on PHC.

The term “primary health care” was first used in the 1920s by the U.K. government in the white paper called the Dawson Report. The report suggested that health care centres should become a model for providing community health services and respond to the increasing complexity of health care as a strategy for tackling health inequalities [9].

Participants in the International Conference on Primary Health Care, held in September 1978 in Alma, declared that:

Primary health care is essential health care based on practical, scientifically sound and socially acceptable methods and technology made universally accessible to individuals and families in the community through their full participation and at a cost that the community and country can afford to maintain at every stage of their development in the spirit of self-reliance and self-determination. It forms an integral part both of the country’s health system, of which it is the central function and main focus, and of the overall social and economic development of the community. It is the first level of contact of individuals, the family and community with the national health system bringing health care as close as possible to where people live and work and constitutes the first element of a continuing health care process.

[10]

In October 2018 (Alma–Ata 40th Anniversary), the Declaration on Health Care and its vision for the future signed by Astana Global Conference on Primary Health Care reinforced the commitment of countries and international partners to make concerted efforts to head health care systems in the direction of primary health care and move towards universal health coverage and health-related sustainable development goals (SDG 3). The vision of 21st century primary health care suggests a comprehensive approach, emphasizing three components [8]:

- Meeting people’s health needs through comprehensive promotive, protective, preventive, curative, rehabilitative, and palliative care throughout the life course, strategically prioritizing key health care services aimed at individuals and families through primary health care and the population through public health functions as the central elements of integrated health services;
- Systematically addressing the broader determinants of health (including social, economic and environmental factors, as well as individual characteristics and behaviour) through evidence-informed policies and actions across all sectors;
- Empowering individuals, families, and communities to optimize their health, as advocates for policies that promote and protect health and well-being, as co-developers of health and social services, and as self-carers and caregivers.

According to the 2014 synthesis of Hungary’s National Institute for Quality and Organizational Development in Health Care and Medicines, primary health care is the first level for people to meet the health care system and to indicate their health problems, where they can be met in most of their prevention and healing needs. The main functions of primary health care are prevention and screening, diagnosis, triad activity, referral to specialist care, coordination of care for chronic patients, treatment of episodic diseases, and palliative care (i.e., improving the quality of life of patients with severe or end-stage diseases). Effective primary health care is holistic, health- (and not disease-) centred [11].

According to Kringos (2013) [12], primary health care can be defined and approached as a multidimensional concept. The strength of primary health care is determined by 10 main dimensions in the following three grouping categories: the structure, process (service provision), and outcome of the care (Table 1).

The above-mentioned elements and Starfield’s (1998) [13] 4C categorization (contact, continuous, comprehensive, coordination) are combined in the definition of primary health care provided by the European Commission’s Expert Panel (EXPH):

Primary health care is the provision of universally accessible, integrated person-centred, comprehensive health and community services provided by a team of professionals accountable for addressing a large majority of personal health needs. These services are delivered in a sustained partnership with patients and informal caregivers, in the context of family and community, and play a central role in the overall coordination and continuity of people’s care.

[14]

There is strong evidence that primary health care can bring many economic benefits in terms of increasing health outcomes, efficiency in health systems, and equity in health.

- Health outcomes—primary health care can improve population health in terms of life expectancy, all-cause mortality [15,16], maternal, infant and neonatal mortality [17,18] as well as mental health outcomes [19,20].
- Health system efficiency—primary health care can reduce total hospitalizations [21,22], avoidable admissions [23,24], and emergency admissions and hospitalizations [25,26].
- Health equity—primary health care improves equitable access to health care [27,28,29] and equitable health outcomes [30,31].

In 2014, KPMG–Nuffield Trust’s analysis of supply and demand conditions for primary health care highlighted the following factors as sources of emerging needs and challenges that increase health care spending: increasing patient expectations, aging populations, new technologies and treatments, increasing incidence of chronic diseases and multimorbidity, demand risen by new service providers, and lack of access to social care. In countries where the traditional model of primary health care is operating, the satisfaction of such needs may be hampered by a number of factors, such as the small independent practices, inadequate and short-term consultations, missing up-to-date communication tools, the lack of adequate diagnostic support, the inadequate connection to specialist care, the lack of meaningful role in overall care coordination, or even pay mechanisms which are negatively incentive [32].

The basic problem of the research presented in this paper is the territorial inequalities of access to primary health care, including the access to general practitioner and paediatric care. Referring to a wide range of literature sources, of the factors outlined in Table 1 which determine the strengths of primary health care, Kringos (2013) has identified seven characteristics of access to care:

1. *Availability of primary health care services:* The volume and type of primary health care services relative to population needs,
2. *Geographic accessibility of primary health care services:* Remoteness of services in terms of travel distance for patients,
3. *Accommodation of accessibility:* The manner in which resources are organized to accommodate access (e.g., appointment system, after-hours care arrangements, home visits),
4. *Affordability of primary health care services:* Financial barriers patients experience to receive primary health care services, such as co-payments and cost-sharing arrangements,
5. *Acceptability of primary health care services:* Patient satisfaction with the organization of primary health care,
6. *Utilisation of primary health care services:* Actual consumption of primary health care services,
7. *Equality in access:* The extent to which access to primary health care services is provided on the basis of health needs, without systematic differences on the basis of individual or social characteristics [12].

In Hungary, the general practitioner-based primary health care system based on the patient’s long-term choice, with corrected capitation-type financing was launched in 1992, and the government tried to contribute to the attractiveness and recognition of the profession with a 50 percent wage increase as part of the primary health care reform. The ability of the system to develop further was greatly limited by the fact that, despite the free choice of doctors, there was no quality competition induced between service providers and it did not develop a preventive and caregiving competence for primary health care. Many conflicts arose from the assignment of primary health care tasks to municipalities, and, consequently, from the constraint of purchasing practice right. Today, the general practitioner staff is aging, suffering from emigration (which cannot be offset by the output of training), and is unable to cope with the challenges of growing expectations and rapidly evolving technologies [33].

In 2015, the working document of the State Secretariat of Health in the Ministry of Human Resources, responsible for health care, “The Concept of Strengthening Primary Health Care” considers the gatekeeper role of general practitioner care as a priority related to the development of primary health care:

The general practitioner plays a significant role both in terms of the role of full care and as a key player in patient management. The general practitioner is the first meeting point for patients with the health care system, and, with the exception of some special care exceptions, the patients can only go to other areas of health care with a general practitioner referral. Gatekeeper function is equally important for quality and effective patient care, especially when it comes to waiting for queues in specialist care and the resources are not available to satisfy the needs.

([34], p. 10)

### 1.1. The Fundamentals of the Legislation of Primary Health Care in Hungary

Article XX, paragraph 1 of the Fundamental Law of Hungary [35] declares that “everyone has the right to physical and mental health”. Under paragraph 2, Hungary promotes the enforcement of this right by, inter alia, organizing health care. According to section 1 (1) of the CXXIII/2015 Primary Health Care Act [36], this form of care ensures that the patient receives long-term, personalized, continuous health care at the place of residence, based on his or her choice, regardless of gender, age, and the type of illness. The statutory areas of primary care are: general practitioner and family paediatric care; dental care; childcare officer care; on-duty care related to primary care; school health care; home care connected to primary care and home hospice care; occupational health care.

“Territorial care obligation” is the mandatory task of the owner, the manager, and the provider of health care services to deliver service to the ones who are entitled to it based on compulsory health insurance, by using the reserved care capacities in the defined care area, as it is declared in the § 1/1/n of the Act CXXXII of 2006 on the development of the health care system [37]. According to the § 13/4 of the local government act (CLXXXIX of 2011), the provision of primary health care services is the obligation of the local government, while the county and the capital’s self-government has to provide specialized care [38].

Pursuant to the 1st paragraph of section 8 of the Primary Health Care act [36], the general practitioner (hereinafter: GP) provides personal and continuous care aiming for the protection of health, prevention, early recognition and treatment of diseases, and health promotion. According to the 2nd paragraph of the same section, the family paediatrician provides the care described in paragraph 1 for persons under 19 years of age. For patients between 14 and 19 years of age, child doctor care can be provided by the GP, based on the choice of the patient to do so [36].

Depending on the structure of the settlement, GP activity can be organized into a ’child district’ (primary health care for people under 14 years of age), ’adult district’ (for over 14-year-olds), or a ’mixed district’ (without age restrictions for the entire population). According to the law—besides territorial restrictions—the free choice of GP is a fundamental right of every citizen.

GP activity can be performed in a public servant relationship with a local government or a health care institution carrying out local government tasks, or in the framework of an individual or social enterprise, as it is written in the 4/2000. (II. 25.) Minister of Health Decree on general practitioner, paediatric, and dental care [39].

### 1.2. Criteria of the Establishment of General Practitioner and Family Paediatrician Districts

In accordance with the provisions of the Primary Health Care Act [36], general practitioner districts have to be designed so that the GP can be reached within 15 min from the outermost point of the district by public transport or on foot, and that the population in the area safely reaches the minimum funding level, but does not exceed the maximum number of people that can be accepted from the professional point of view, and the personal and material conditions of general practitioner care must be provided. As it is declared in the Government Decree 43 of 1999 on the detailed rules for financing health services from the Health Insurance Fund [40], financing agreement for a new adult or mixed general practitioner service is available for a population of 1200–1500 over 14 years, while in the case of child districts, the funding range is 600–800 children under 14 years of age. Of course, this funding minimum does not apply to already operating districts.

Pursuant to the 4/2000. (II. 25.) Minister of Health Decree, to every adult and mixed GP district, at least one, so-called ‘district nurse’ belongs, who participates in the general practice management, prevention, screening, nursing, health education and advisory activities, manages administration, carries out the maintenance of medical tools and devices, provides first aid and organizes emergency care when needed, and co-operates with the childcare officer and the actors of the social care system [39].

### 1.3. The Permanently Vacant General Practitioner and Family Paediatrician Districts

As stated in the Government Decree 313 of 2011. (XII. 23.) [41], the general practitioner district with territorial care obligation, in which the obligation can be met by only substitution for more than six months, unless the reason for this is that the general practitioner of the district is disabled or in which—with the exception of substitution—the municipality cannot provide the person who is entitled to perform an independent medical activity for at least six months.

According to Minister of Health Decree 4 of 2000. (II. 25.) [39], a physician who, having possession of an approved individual training plan for obtaining the required practice, has commenced specialist training, until obtaining the right to pursue an independent general practitioner activity, but for up to six years may perform in the permanently vacant general practitioner district as a substitute general practitioner (except paediatrician), for the benefit of the territorial care certified by the local government. In this case, the substitute physician is in the position of a civil servant with the Office of the Chief Medical Officer of State and performs the duties of general practitioner in the district designated by the Office, but the agency concludes a contract with the local government.

### 1.4. Register of Practice Rights

Act II of 2000 on the substantive medical activity [42] and the Government Decree 313/2011 (XII. 23) on its implementation [41] declares that the condition of individual general practitioner activity is legal force of the decision granting the property right called ’practice right’. The practice right is a license issued by the health administration (the health institutes of district Government Offices), which entitles the person concerned to pursue an independent general practitioner activity in the district designated by the municipality. The practice right can be disposed of and can be continued under certain conditions, but it may not be leased. As of 1 January 2012, the right to operate as a general practitioner working with the territorial supply obligation is considered as practice right. 

Financing the contract for the performance of general practitioner tasks, operation of general practitioner service, and remuneration can be concluded in the relevant regional offices of the National Health Insurance Fund of Hungary (hereinafter: NHIF). The contract with the municipality is required for the conclusion of the contract and the valid and applicable operating license. After the operation of general practitioner services, the GPs may receive a monthly financing fee on the following items: fixed fee, territorial supplementary remuneration, performance-based remuneration, ad hoc fee, remuneration for controlling legal relationships, remuneration for the results achieved in the indicator system, additional remuneration for staff, remuneration for overhead costs.

### 1.5. The Measures Aiming the Occupation of Vacant Districts

In a permanently vacant adult or mixed district, doctors who do not have yet a general practitioner examination may be substitutes in the framework of the Praxis I program, as an employee of the National Healthcare Services Centre (hereinafter: NHSC) concluding the contract of assignment. The exam must be obtained by the doctor concerned within a maximum of six years, and, during this period they work four days per week, and, in the rest of the time they must complete their clinical practice in an accredited department of a nearby hospital. The Praxis II program enables doctors with clinical qualifications in the given professions to perform general practitioner activities in an adult or mixed district with territorial care obligation. In order to start general practitioner activity, the physician must complete the training plan and the general medical examination within five years for a valid operating license, an approved individual training plan, the acquisition of practice right, a contract for the performance of tasks with the local government, a training contract concluded with the NHSC, a financing contract and a successful social security knowledge exam.

Pursuant to section 18/A paragraph 1 of the 43/1999. (III. 3.) Government Decree [40], in the framework of the National Health Insurance Fund’s 2019 call for applications for the resettlement of the doctors occupying the permanently vacant general practitioner (…) districts, the doctor who is entitled to perform general practitioner/dentistry activities on the basis of the personal conditions specified in the 4/2000. (II. 25.) Minister of Health Decree [39], and who undertakes that he/she will perform medical activity in the particular general practitioner district for at least six years in a form of operation based on the contract concluded with the competent local government, is entitled to a grant of HUF 20 million in five different categories, based on the length of the district’s vacancy. The population to be cared by the general practitioner must exceed 1000 people in the case of an adult and mixed district, and 500 in the case of child districts.

Pursuant to paragraph 2 of the same section of the above-mentioned decree, NHIF grants general practitioners without practice right a non-refundable subsidy for starting/restarting the practice, replacing physicians wishing to sell their practice, in order to reduce the number of vacancies, or at least not to let it increase, the continuous medical care of the population to be provided so that the general practitioner will operate at a higher standard. The doctor who did not have the right to practice as a general practitioner in the two years preceding the announcement of the application may submit an application on the basis of the personal conditions specified in the 4/2000 (II. 25) Minister of Health Decree [39], and undertakes to perform independent medical activities in the given general practitioner service for at least four years in the form of an operating agreement with the relevant local government, but in all cases with personal assistance. The population to be taken care of by the general practitioners who may be entitled to the acquired practice should exceed 800 people in the case of an adult and mixed district, and 400 in the case of child district.

## 2. Materials and Methods

For the analysis of the situation of permanently vacant general practitioner and family paediatrician districts, the *December 2018 data of the register maintained by the Primary health care Department of the National Healthcare Services Centre* was taken into account. The spatial distribution of the occupied and vacant districts by breakdown by type of district was examined for the child, adult and mixed districts, in a descriptive way.

**Figure 1:**
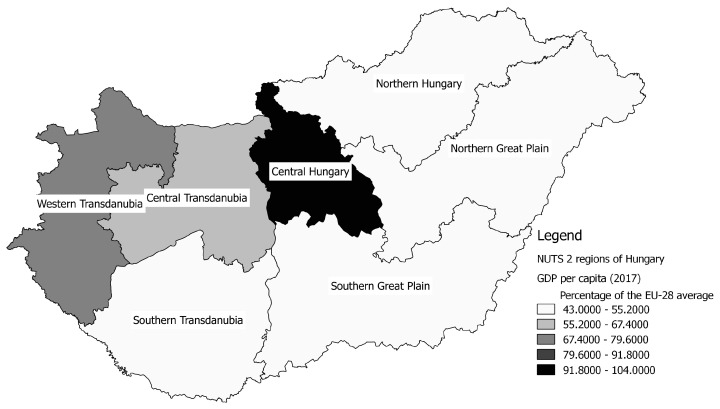
The Hungarian NUTS 2 regions by GDP per capita (as percentage of the EU-28 average), 2017. Source: own editing based on Eurostat data [43,44], 2019.

**Figure 2:**
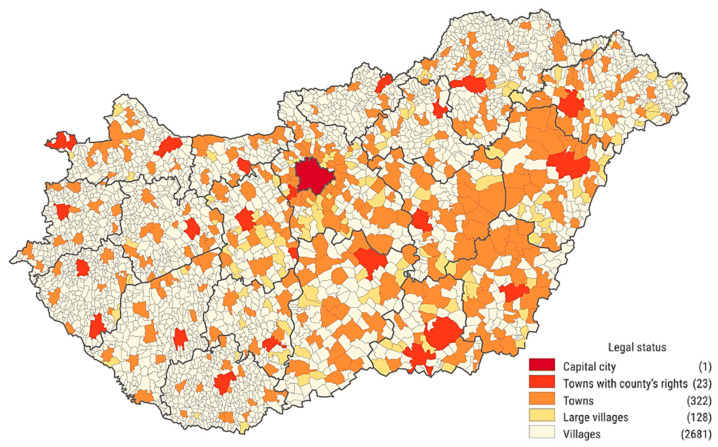
The settlements of Hungary by legal status, 1 January 2019. Source: Hungarian Central Statistical Office (edited) [45].

In order to study the truly relevant relationships among the characteristics of the permanently vacant GP districts, mathematical statistical analysis was performed. First, the relationships between the variables interpreted on the categorical measurement scale were examined in order to substantiate the more general statements. For this the method of crosstab analysis was used. The variables and their established categories were:

- Region: NUTS* 2 level administrative region comprising the municipality being the centre of the district (see the regions on Figure 1 with the classification of GDP/capita value) * Nomenclature of Territorial Units for Statistics (Eurostat)
- Type of general practice: Adult district; Child district; Mixed district
- Size class of settlement: 0 to 499; 500 to 999; 1000 to 1999; 2000 to 4999; 5000 to 9999; 10,000 to 19,999; 20,000 to 49,999; 50,000 to 99,999; 100,000 people or more (read more in Box 1, see Figure 2)
- Suitability of district population size (based on Government Decree 43 of 1999 [40]): Properly sized; Undersized; Oversized (Adult and mixed districts: min. 1200, max. 1500 people; Child districts: min. 600, max. 800 people)
- Length of vacancy: Less than 1 year; 1 to 5 years; 6 to 10 years; More than 10 years

Box 1 **Description of the spatial structure of Hungary. Source: own editing based on the data of Hungarian Central Statistical Office, 2019.**

In Hungary there are 3155 settlements, which are aggregated in 197 sub-regions, 19 counties, 8 (formerly 7) regions and 3 macroregions. The population of the country was 9,945,475 on 1 January 2019. Of the 346 towns 23 have county’s rights. The number of villages is 2681, while the category of large villages with the population above 5000 people counts 128 settlements. 92 percent of the Hungarian settlements fall into the size category under 5000 inhabitants, which makes 32% of the total population. The proportion of settlements populated by less than 1000 residents is 57 percent, where 8% of the Hungarian population live. Most of the small villages can be found in the Western and Southern Transdanubia, and, in Northern Hungary, while the Northern and Eastern Great Plain can be characterized by fewer larger settlements.

The significance of associative relationships was investigated by Pearson’s Chi-square test (hereinafter χ^2^). The symmetric Cramer’s V (φ_c_) was used to determine the relationship between the nominal variables in each case. The indicator measuring the strength of the relationship between the nominal variables ranges from 0 to 1.

For the better understanding of the relationships of each district type (child, adult, mixed), the correlation between the variables interpreted on the metric measurement scale was analysed. For each type of district, the co-movement of the following ratio variables was examined:

- POP_CENT: Population of the settlement where the district centre is located (capita)
- POP_DIST: Total population to be served in the district (capita)
- PROP_AGE: Proportion of total population to be served in the district in relation to the concerned age group of the municipality in which the district centre is located (%)
- VAC_LENGTH: District vacancy length (years)

In order to ascertain the suitability of the variables for calculating the Pearson linear correlation coefficient, normality test was performed. Given that both the Kolmogorov–Smirnov test and the Shapiro–Wilk test were significant (*p* ≤ 0.01) for all districts [46], as well as the scatterplot diagrams proved the lack of normality, it was obvious that the variables included in the study did not follow the normal distribution, so the linear correlation calculation cannot be performed on them. Instead, rank correlation was used that is less sophisticated, but insensitive to outliers and distribution. It measures the direction and strength of the relationship between ordinal variables (rankings) generated by the order of the original metric. The Spearman coefficient ρ (hereinafter r_s_) is interpreted as falling between −1 and 1.

In the case of Cramer’s V, the strength of the relationship was interpreted as follows [47]: <0.05, no or very weak; 0.05–0.1, weak; 0.1–0.15, moderate; 0.15–0.25, strong; >0.25, very strong.

For rank correlation, based on the values obtained—when there was a significant relationship—the following intervals were used [48]: <0.2, weak; 0.2–0.4, weak to moderate; 0.4–0.7, strong medium; >0.7, strong.

The study provides an overview on the government measures for the sake of solving the occurrence of vacancy and improving access in the lagging regions and settlements. To get a deeper insight into the development of vacant districts, a semi-structured interview was conducted with staff at the Primary Health Care Department of the National Health Care Services Center. The relevant statements of the conversation on the basic situation and broader context of the GP system are presented in this article after the statistical analyses are detailed.

## 3. Results

### 3.1. Territorial Distribution of the General Practitioner and Family Paediatrician Districts

In December 2018 there were 1492 child, 3365 adult, and 1486 mixed general practitioner districts with territorial care obligation in Hungary. One third of the child districts and 37.77% of adult districts are located in the Central Hungary region, while 20.86% of the mixed districts are in Northern Hungary. The centres of child and adult districts have a larger proportion in the settlements in larger population categories, while the majority of mixed district centres (89.09%) are concentrated in settlements with more than 500 but fewer than 5000 people. Conversely, 92.83% of the district centres that can be found in the settlement categories below 2000 inhabitants supply mixed districts, in the settlements with the population of 2000 to 4999, 46.49% provide care for mixed, 35.14% for adult, and 18.38% for child district. Over 5000 people, to 69.13% of the district centres an adult district belongs, 29.95% carry a child district, and only 0.92% a mixed district.

### 3.2. Data on the Vacant Districts

**Figure 3:**
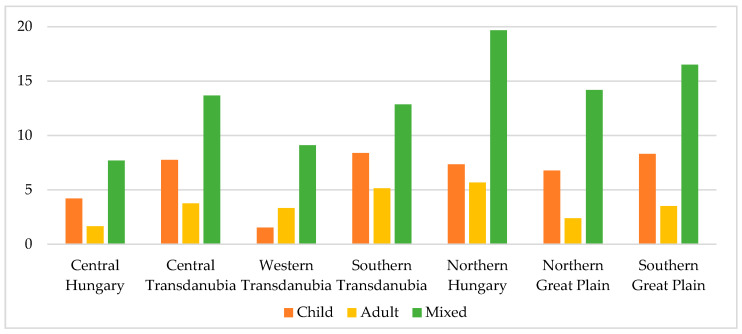
The proportion of vacant districts in the Hungarian NUTS 2 regions by the type of district (%), December 2018. Source: own editing based on the data of NHIF, 2019.

In December 2018, 89 child, 101 adult, and 212 mixed districts formed the set of vacant districts, which means that 5.97% of child districts, 3.03% of adult districts and 14.27% of mixed districts with territorial care obligation were vacant. In the case of child districts, the vacancy rate was highest in Southern Transdanubia (8.39%), the lowest in Western Transdanubia (1.53%). The proportion of vacant adult districts was the biggest in Northern Hungary (5.67%), the smallest (1.65%) in Central Hungary, and the same two regions represented extreme values in proportion to vacant mixed districts (19.68% and 7.69%) (see Figure 3).

At the national level, most of the vacant child districts belong to the district centres located in settlements with 2000–4999 inhabitants and 10,000–19,999 inhabitants, both with a proportion of 23.6 percent, while 13.48% is the share of vacant child districts with their centre in settlements with a population of 5000–9999 and 50,000–99,999 as well. One third of the vacant adult district centres are located in settlements with 2000–4999 inhabitants, but small towns with a population of 10,000–19,999 contribute to the set of settlements being centres of vacant adult districts to the extent of 16.67%, and the ones with a population of 5000–9999 to the extent of 15.69%. A total of 37.74% of the vacant mixed districts can be found in the settlements with 500–999 inhabitants and 33.02% in the settlement category with the population of 1000–1999.

**Figure 4:**
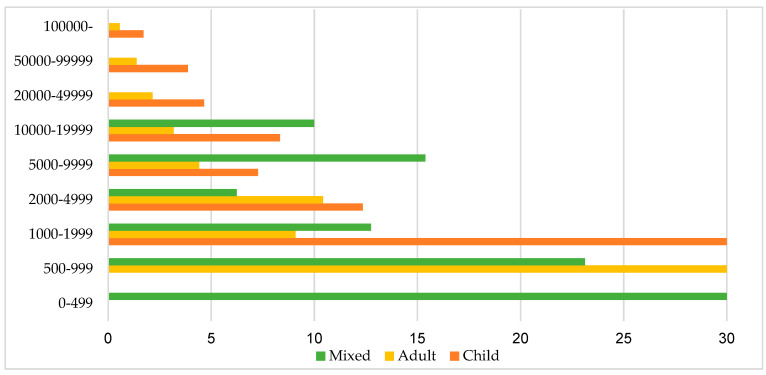
The proportion of vacant district centres in different settlement size categories by the type of district (%), December 2018. Source: own editing based on the data of NHIF 2019.

In the population categorization, the most important corner numbers are as follows: 30% of mixed districts with a centre in villages of 0–499 people are vacant, and the similar is the proportion of centres of vacant adult districts in villages with 500–999 inhabitants, as well as the share of vacant child district centres among the settlements populated by 1000–1999 people. It should also be noted that 15.39% of mixed districts in settlements with 5000–9999 inhabitants are vacant (see Figure 4).

According to the length of long-term vacancy, in December 2018, the oldest vacant districts had no permanent general practitioner for the past 14 years. In 70.79% of vacant child districts the general practitioner’s practice became vacant three years or earlier, in 70.3% of adult districts within one year, while in 72.17% of mixed districts for five years or earlier. In the case of mixed districts, it should be remarked that 14.62% of the practices are vacant for 10 years or more, while the same length of vacancy is characteristic for 3.96% of the adult, and for 4.49% of the child districts.

Taking into account the population of the districts, it can be stated that the number of patients affected by the vacant districts of a child, adult, or mixed type of care is more than half a million (exactly 510,193 people, based on the examined statistics), of which 60,219 is the child, 172,309 the adult, and 277,665 the mixed vacant districts’ total population supplied. Regarding all types of care, based on the December 2018 data, 349,729 people, 66.55% of the population concerned by the vacancy of districts, belong to a district centre settlement with a population fewer than 5000 inhabitants.

Regarding the distribution of the population supplied, and, taking into account the relevant provisions of the aforementioned Government Decree 43/1999 [41], the adult and mixed districts with a supplied population of 1200 to 1500 people are considered to be of the appropriate size, while those below this range are undersized and above this range oversized. In child districts, the compliance interval is 600–800 according to the above-mentioned legislation. 68.32% of the vacant adult districts are oversized, so the typical type of district in larger settlements is usually more heavily loaded than it should be, while the proportion of undersized vacant adult districts is only 7.92%. The share of the different categories is more balanced in mixed vacant districts, but the proportion of undersized districts is 44.13%, while more than one third is oversized. With regard to the occurrence of mixed districts according to the size of settlements, it is not surprising that in many cases the district covering several small settlements does not reach the lower threshold of compliance. Even in the case of vacant child districts, the undersized ones represent the highest proportion (42.7%), but the one-third proportion of the districts with the proper number of patients exceeds the 23.6% share of the oversized ones in this type of district.

### 3.3. Interrelations of the District Vacancies

The distribution of settlements by population size in Hungary is not uniform. In the case of Northern Hungary and Western and Southern Transdanubia, the proportion of small villages dominates, in the Northern and Southern Great Plain the proportion of large villages and small towns is dominant, while in Central Hungary the role of small- and medium-sized towns is significant. This is also more or less reflected in the territorial distribution of settlements providing centres for vacant districts. The cross-tabulation analysis also confirms a very intense association between the size of the settlement and the type of general practitioner service already revealed by the descriptive analysis (χ^2^(16) = 262.097, *p* < 0.01; φ_c_ = 0.628, *p* < 0.01)—in smaller settlements, mixed districts are predominant in their proportion, and these areas are most affected by vacant status.

The number of inhabitants to be served in a given district is also significant in relation to the size of the settlement being the centre of the district (χ^2^(16) = 97.394, *p* < 0.01; φ_c_ = 0.442, *p* < 0.01). Vacant districts with settlement centres of fewer than 1000 inhabitants are typically undersized. Overpopulation dominates in the case of settlement centres with the population between 1000 and 10,000. In settlements with 10,000 to 19,999 inhabitants there are slightly more vacant districts with adequate load than those with oversized districts. Between 20,000 and 49,999 inhabitants, the number of eligible and oversized districts is balanced. In settlements with the population of 50,000 to 99,999, the number of oversized vacant districts is more than twice the amount of the corresponding ones. With over 100,000 people, the number of vacant districts with a sufficient load exceeds that of the oversized, and there is no longer an undersized district in this population size category. The number of undersized residual districts and the proportion of vacant districts are highest in the Southern Great Plain, while the proportion of oversized districts is highest in Northern Hungary. In the Central Hungary region, the amount of oversized vacant districts is more than the number of properly sized ones and undersized ones combined.

There is a very strong relationship between the statistical region including the central settlement of the district and the length of the district vacancy (χ^2^(18) = 49.978, *p* < 0.01; φ_c_ = 0.333, *p* < 0.01). In the regions of Northern Hungary, south Transdanubia, Northern and Southern Great Plain, which are less favourable in terms of their development and socio-economic status, most of the districts have been vacant for one to five years, and, most of the districts that have been vacant for more than 10 years can be found in these regions, while the newly vacant districts dominate in the regions of Central Hungary and the central and Western Transdanubia.

The calculation of rank correlation over the entire set of permanently vacant districts, irrespective of their type, had relevance for a single data pair: the population of the district-centre settlement has a significant (*p* < 0.01), opposite, weak–moderate correlation (r_s_ = −0.335) with the length of long-term vacancy. Cross-tabulation analysis shows an association with similar intensities for the above characteristics for the entire group of vacant districts: in the settlement categories below the population of 2000, most districts became vacant in the five years preceding 2018, similar to the settlements populated with more than 5000 but fewer than 100,000 inhabitants. In the settlements with 2000 to 4999 residents and over 100,000 inhabitants, the districts that became vacant in 2018 form the majority. Most vacancies over 10 years are centred in settlements with fewer than 1000 inhabitants.

Keeping the logic of descriptive analysis, the relationships of the individual variables by district type were examined in order to draw specific conclusions for children, adult, and mixed districts regarding region, settlement size, population served, adequacy of district size, and length of vacancy (see Table 2).

In the child districts, there is a significant, weak–moderate relationship between the population of the district centre settlement and the population aged 0–14 supplied in the district (r_s_ = 0.364, *p* < 0.01). Thus, the potential load of the vacant child districts by the concerned age group of the settlement is expected to be higher in the larger settlements. This is much more pronounced in mixed districts, where there is a significant, medium-strong relationship between the population of the settlement being the centre of the district and the population served by the district (r_s_ = 0.594, *p* < 0.01). There is no statistically significant correlation between these parameters in adult districts (*p* = 0.782).

There is a very strong negative correlation between the population of the district centre settlement and the proportion of the population served in the entire district compared to the concerned age group of the central settlement in the vacant child (r_s_ = −0.968, *p* < 0.01) and adult (r_s_ = −0.977, *p* < 0.01) districts, and a moderate negative relationship (r_s_ = −0.683, *p* < 0.01) in the mixed districts. While in the case of districts with smaller settlements, the population of the given age group in the district consists of the patients from several surrounding settlements, and thus the supplied population of the central settlement represents only a part of the whole group patients, at the same time, in a large settlement size dimension, a given district can provide care for only a part of the population of the settlement in the particular age group.

The empirical significance level falls below 0.05 in the vacant child districts and below 0.01 in the mixed districts, and an opposite, weak average relationship was found between the population of the settlement being the centre of the district and the length of the district’s vacancy, so the districts consisting of smaller settlements are more likely to be vacant for a long time. In adult districts, this relation cannot be detected based on rank correlation.

The analysis revealed a significant, negative, weak–moderate relationship between the population size and the length of vacancy in the adult districts (r_s_ = −0.284, *p* < 0.01) and a moderate–strong correlation (r_s_ = −0.458, *p* < 0.01) in the case of mixed districts. Given that the common feature in the adult and mixed districts is the provision of care for the entire population over the age of 14, it can be concluded that the districts that are more heavily loaded with the adult population to be served tend to become vacant somewhat later.

### 3.4. Findings of the Expert Interview

According to the experts of the Primary Health Care Department of the Health Services and the Organization Department of the National Health Care Services Centre, the system of general practitioners established in 1992 brought a change of approach; while before, the general practitioner was considered as a role-player at the bottom of the health care hierarchy, after this turn, the primary health care doctor appeared in the public opinion as the “customer of the specialized services”, or the “attorney of the patient”, with the most information about the patient, who knows the care system, and manages the patient’s health. In an optimal case, the services they provide cover prevention, health assessment, referral, and care.

In terms of GP districts, it is essential that it is financially worthwhile for the physician in terms of the size of the area to be treated. Although, for two decades now, the size of the district to be financed was legally determined, there are many residual districts with a low number of inhabitants. It all depends on the geographical conditions, there are small village areas where ten villages make up 1000 inhabitants. Nowadays, the practice of general practitioner can be made attractive mainly by the relative freedom, institutional independence, continuous personal relationship with the patients, and the increasing income level. Praxis I and Praxis II programs have been going on for years, which provide a practical framework for becoming a general practitioner or retraining, and the state supports tenders for the purchase of practice rights and settling down. Experience shows that internship programs are more effective in practical training for GPs than universities are.

In the temporary or permanent occupation of the districts, the settlements that are easier and faster to reach from Budapest or the county seats are more attractive. At the regional level, Budapest, Pest County, and western Hungary are the most popular destinations, while, it is harder to find a doctor in Northern Hungary. Resettlement in the occupied area is rare and is rather more common among physicians moving abroad. In disadvantaged settlements with segregation, the practice is typically taken up by a local physician. The greatest repulsive force in relation to each district is the vulnerability, the constraint of duty, and the difficult-to-handle population.

It is important to note that the development of permanent vacancy is not a single-channel process, so it is not only the physicians’ affection. In many cases, the behaviour of the municipalities is an obstacle to filling the districts; they do not seek family doctors because substitution solves the care and they can terminate the contract at any time, even without giving reasons (there was already a precedent for dismissing a physician participating in Praxis I). At the time of the interview, there was a doctor in Praxis I who has not found a settlement to start his professional practice for half a year. The delegation of experts to each district should be the responsibility of the Office of the Chief Medical Officer of State, as the municipalities can be considered as laymen on this issue. Replacement would be thought to be a hump on the back of general practitioners, because it requires much more care for patients, more mobility between districts, and much less time for each settlement or patient. However, many GPs make a living out of substitution because there is more funding for more districts—it is better than carrying a single district. In many cases, however, the residents put a pressure on the municipality not to let a substitute doctor go because he/she may be persuaded to prescribe anything (e.g., antidepressants).

The fact that cost-effective size and operation are not the main considerations in the filling of the districts in the situation preserved by the individual municipalities is also proved by the fact that in the history of general practitioners there is no instance of district border modification, however, there was already precedent for disintegration. Given that participation in the Praxis I program does not imply the acquisition of the practice, it therefore does not constitute a commitment. However, it can be attributed to the process of mutual understanding between doctors and municipalities that the doctors participating in the Praxis I program remain in the area for which they were employed after obtaining the specialist examination. Professionals come from across the border, mainly from Ukraine, Romania, and Serbia. Generally speaking, physicians who enter the system tend to calculate resettlement or practice purchase opportunities, taking advantage of the fact that while practice programs temporarily address the lack of physicians, the permanently vacant status of the district still remains, and, because of the continuously increasing length of vacancy the financial support for filling the district is growing.

## 4. Discussion

### 4.1. Experiences from Former Empirical Researches on the Primary Health Care

Since the 1990 change of political regime, all governments have been hesitant to address health—they take advantage of the fact that doctors do not organize strikes under the Hippocratic Oath, but care for the patients under all circumstances [49].

The primary health care medical staff is aging nationwide, and many districts do not have their own physicians, as it is not an attractive alternative for young/beginner doctors [50]. Aging GPs are also part of the aging rural population and will need to be replaced. Increasing the size of the districts is by no means a solution to the problem of vacant posts, as substitution is a heavy burden, which fragments the time of the practitioner, and, because of travelling between the districts, it decreases the efficiency of the service. It is obvious that the problem of district vacancy cannot be solved only by increasing the funding [51].

Fatigue is a common feature of primary health care—it is a mass care that eliminates major illnesses, but in many cases, without the possibility of effective cure. Low pay does not increase doctors’ motivation, there is a lack of professional control, and many patients are losing out on routine healing colleagues, who sometimes understate cases. At lower levels of care, cost-effectiveness is a decisive factor, but keeping the patient’s best interests in mind and economically functioning is a huge responsibility [49]. Primary health care should also be strengthened at device level to make the gateway role more dominant through better diagnoses and treatments to help relieve outpatient care [51].

### 4.2. The Effectiveness of State Programs Addressing District Vacancies

The goal of the announced support programs is to create continuity of general practitioner care by assisting and financially encouraging practitioners to settle in a particular area and to purchase expensive practice law. If we take a look at the list of the winners, while resettlement supports are mainly utilized in the regions and small settlements that are the most affected by the presence of vacant general practitioner districts, practice purchase grants are most likely to be found in the more developed regions and larger urban settlements, which are attractive to beginner general practitioners. Of course, it is very important to maintain family doctor care in these latter regions, too, especially due to the high concentration of population, but due to the nature of the problem of vacant areas, in my opinion, basically resettlement support could provide the answer. However, the statistics show a different situation: the number of vacant (adult and mixed) general practitioner districts nationwide increased by 32.6% between 2014 and 2018 (since the beginning of the state programs). Overall, the supports did not necessarily help the areas most affected by the vacancy of the districts and did not contribute to the reduction of the proportion of the vacant practices.

### 4.3. A Possible Future Scenario for the Sytem of General Practice in Hungary

According to Gyula Kincses, who has been the president of the Hungarian Medical Chamber from November 2019, in a modern health care system the necessary and up-to-date medical knowledge can no longer be owned by a single person, and the necessary advanced medical technology cannot be operated cost-effectively by a single physician. In addition, a physician is no longer able to perform the increased, multifaceted tasks of modern primary health care (community and individual health promotion, general and targeted screening, healing, pathway management, etc.), and many of these tasks should be done by a qualified graduate nurse. Today, without teamwork and advanced care organization, the effectiveness of care cannot be achieved, and the effective implementation of preventive, caring-minded work and the gatekeeper function can only be fulfilled by integrating some specialist tasks into primary health care [33].

In a group practice, a community health centre, physicians and nurses of different qualifications together provide an enhanced level of primary care and health services to a community of 15 to 20 thousand people. Practitioners include a GP, a gynaecologist, and a paediatrician, but there may be other qualified physicians. The group’s competence (knowledge level) is thus higher than that of a single GP, as people with different qualifications work together. They use the tools together, so the instrumentation can be more advanced than in a normal practice, and the more expensive instruments are better utilized and rewarded [52].

A forward-looking model experiment in primary health care is a Swiss–Hungarian Primary Health Care Development Model Program, aiming the establishment of the so-called “practice community”. The fundamental difference between a group practice and a practice community is that, in the practice community, physicians (GPs) of the same knowledge, with the same task and competence, work together to provide common professional and public health background support (physiotherapist, dietitian, health promoter, etc.). The patient’s own doctor is the general practitioner here. In the group practice, on the other hand, doctors with different qualifications provide a higher level of care together, and practitioners do not have the same task. The patient’s GP is the group itself, although the patient has a personal contact and health manager [49]. Traditional primary health care physician tasks are thus integrated into the group practice, but the group practice also incorporates a significant portion of the routine practices of the specialized care. At the same time, specialists in the outpatient care take over tasks from the hospitals. They perform specialized care in day surgery or as a day hospital [33].

The idealistic place of the group practice would be the so-called “microregional health centre”, where the physicians and workers of the practice, as well as the diagnostic and therapeutic facilities, are located, or the clinics/offices hosting external specialists (e.g., ophthalmologist, urologist, dermatologist etc.), and where the patients could be transported, instead of substitute GPs travelling between the run-down consulting rooms of the villages [52].

Kincses [33] also claims that the vision for transformed primary health care cannot be a homogeneous model of care; in the medium term, group practice is expected to be the dominant form of operation, but in addition there will be practice communities, care practices, and the well-functioning (current) individual practices will continue to exist. The number of primary health care points is necessarily decreasing, so it has to be figured out what the basic service of the health care system will be in the future, which must be ensured in every settlement [52].

A major obstacle to the above-described transformation is the institution of practice right (previously detailed in Section 1.4) which, as a property right, may make the participants of the group practices conflicted (because of the GPs having, but the specialist physicians not having, this property right). The re-consideration of the so-called “Practice Fund” would be needed, that purchases the vacated right from the owner of the terminating (and transforming) practices [33].

## 5. Conclusions

The vacant general practitioner and family paediatric districts are continuously appearing in Hungary, which are part of a fragmented, non-efficient operating structure. The aging and migration of the physicians foster this process, and the emptying practices are not attractive for the active GPs, due to the distance, approachability, and the socio-economic and infrastructural situation of the settlements belonging to these districts. Financing is already an issue at the very beginning—one of the great anomalies of the Hungarian legal system is that in order to fill an inherently deficient profession, it is necessary to buy a property right, known as practice right.

In terms of volume and length, the long-term vacancy affects mostly mixed districts, which can mainly be found in smaller settlements, in parts of the country with small villages, and many of them in the Northern Hungary region. In the latter region, most of the vacant districts are oversized compared to the provisioned population interval defined by the law. The frequent occurrence of under- or oversizing in any region, district type, or town-size category is due to the fact that the legislation created in 1999 only regulates the burden of newly created districts, not the old ones, and consequently there are many of the inadequately dimensioned residual districts. Regarding the time of becoming vacant, the shortage of doctors in the last few years has begun to reach first the child, then the adult districts of the larger settlements, and in 2018 this process escalated.

The practice of many municipalities in relation to substitution does not help to solve the problem of vacancy. Many substitute GPs also builds an existence on the additional funding. Public programs are effective by deploying professionals being trained in vacant districts, and by supporting practice buying and resettlement, but are not able to counteract the pace of aging and emigration and seek to preserve an outdated system. The behaviour of local decision makers sometimes overwrites program objectives too.

The author agrees with Gyula Kincses that the Hungarian primary health care system of the future should not be unified, but the combination of group practices with multiple specializations, practice communities with the co-operation of GPs and the traditional individual practices. The introduction of the new forms of family practitioner care must be an evolutive process, with regard to the spatial structure and the needs of the different areas, and the co-operation of physicians in the creation of practice groups and communities should be a voluntary action supported by fundamental changes in the system of practice rights.

## Figures and Tables

**Table 1:** Dimensions of the strength of primary health care. Source: own editing based on Kringos (2013).

Structure	Process	Outcome
Governance Economic conditions Workforce development	Access Continuity Coordination Comprehensiveness	Quality Efficiency Equity

**Table 2:** Value, direction, and significance of the Spearman correlation for metric variable pairs by the type of district. Source: own calculation based on the data of NHIF, 2019.

Spearman’s ρ			POP_CENT	POP_DIST	PROP_AGE	VAC_LENGTH
**CHILD vacant districts** **N = 89**	**POP_CENT**	r_s_	1.000	0.364 **	−0.968 **	−0.219 *
		*p (2-sided)*	.	*0.000*	*0.000*	*0.039*
	**POP_DIST**	r_s_	0.364 **	1.000	−0.183	−0.156
		*p (2-sided)*	*0.000*	.	*0.086*	*0.145*
	**PROP_AGE**	r_s_	−0.968 **	−0.183	1.000	0.215 *
		*p (2-sided)*	*0.000*	*0.086*	.	*0.043*
	**VAC_LENGTH**	r_s_	−0.219 *	−0.156	0.215 *	1.000
		*p (2-sided)*	*0.039*	*0.145*	*0.043*	.
**ADULT vacant districts** **N = 101**	**POP_CENT**	r_s_	1.000	−0.028	−0.977 **	−0.081
		*p (2-sided)*	.	*0.782*	*0.000*	*0.422*
	**POP_DIST**	r_s_	−0.028	1.000	0.187	−0.284 **
		*p (2-sided)*	*0.782*	.	*0.062*	*0.004*
	**PROP_AGE**	r_s_	−0.977 **	0.187	1.000	0.023
		*p (2-sided)*	*0.000*	*0.062*	.	*0.817*
	**VAC_LENGTH**	r_s_	−0.081	−0.284 **	0.023	1.000
		*p (2-sided)*	*0.422*	*0.004*	*0.817*	.
**MIXED vacant districts** **N = 212**	**POP_CENT**	r_s_	1.000	0.594 **	−0.683 **	−0.290 **
		*p (2-sided)*	.	*0.000*	*0.000*	*0.000*
	**POP_DIST**	r_s_	0.594 **	1.000	0.033	−0.458 **
		*p (2-sided)*	*0.000*	.	*0.638*	*0.000*
	**PROP_AGE**	r_s_	−0.683 **	0.033	1.000	0.064
		*p (2-sided)*	*0.000*	*0.638*	.	*0.352*
	**VAC_LENGTH**	r_s_	−0.290 **	−0.458 **	0.064	1.000
		*p (2-sided)*	*0.000*	*0.000*	*0.352*	.

* Correlation is significant at 5% level (two-sided). ** Correlation is significant at 1% level (two-sided).
